# Expression Profiles and Potential Functions of Long Non-Coding RNAs in the Heart of Mice With Coxsackie B3 Virus-Induced Myocarditis

**DOI:** 10.3389/fcimb.2021.704919

**Published:** 2021-08-24

**Authors:** Xiang Nie, Huihui Li, Jin Wang, Yuanyuan Cai, Jiahui Fan, Beibei Dai, Chen Chen, Dao Wen Wang

**Affiliations:** Division of Cardiology and Hubei Key Laboratory of Genetics and Molecular Mechanisms of Cardiological Disorders, Tongji Hospital, Tongji Medical College, Huazhong University of Science and Technology, Wuhan, China

**Keywords:** acute myocarditis, fulminant myocarditis, lncRNAs, inflammation, coxsackie B3 virus

## Abstract

**Aims:**

Long non-coding RNAs (lncRNAs) are critical regulators of viral infection and inflammatory responses. However, the roles of lncRNAs in acute myocarditis (AM), especially fulminant myocarditis (FM), remain unclear.

**Methods:**

FM and non-fulminant myocarditis (NFM) were induced by coxsackie B3 virus (CVB3) in different mouse strains. Then, the expression profiles of the lncRNAs in the heart tissues were detected by sequencing. Finally, the patterns were analyzed by Pearson/Spearman rank correlation, Kyoto Encyclopedia of Genes and Genomes, and Cytoscape 3.7.

**Results:**

First, 1,216, 983, 1,606, and 2,459 differentially expressed lncRNAs were identified in CVB3-treated A/J, C57BL/6, BALB/c, and C3H mice with myocarditis, respectively. Among them, 88 lncRNAs were commonly dysregulated in all four models. Quantitative real-time polymerase chain reaction analyses further confirmed that four out of the top six commonly dysregulated lncRNAs were upregulated in all four models. Moreover, the levels of ENSMUST00000188819, ENSMUST00000199139, and ENSMUST00000222401 were significantly elevated in the heart and spleen and correlated with the severity of cardiac inflammatory infiltration. Meanwhile, 923 FM-specific dysregulated lncRNAs were detected, among which the levels of MSTRG.26098.49, MSTRG.31307.11, MSTRG.31357.2, and MSTRG.32881.28 were highly correlated with LVEF.

**Conclusion:**

Expression of lncRNAs is significantly dysregulated in acute myocarditis, which may play different roles in the progression of AM.

## Introduction

Acute myocarditis (AM), including fulminant myocarditis (FM) and non-fulminant myocarditis (NFM), is an inflammatory disease of the heart muscle with variable clinical presentations. AM can be caused by a broad range of infectious agents, including viruses, bacteria, fungi, and protozoa, as well as non-infectious triggers, such as toxins and hypersensitive reactions (Sagar et al., 2012; Fung et al., 2016). Among these triggers, viral infection (especially coxsackie B3 virus, CVB3) has been recognized as the most common cause of myocarditis (Fairweather et al., 2012; Sagar et al., 2012). Pathological changes in the virus-infected myocardium have been well recognized, ranging from viral replication, activation of innate and acquired immune responses, to virus-induced cardiac damage and dysfunction (Shirani et al., 1993; Caforio et al., 2013). Pathogens and immunological responses can cause acute structural and functional abnormalities in the heart, which in turn leads to regional or global cardiac contractile impairment. Advances in cardiac magnetic resonance imaging (cMRI) and endo-myocardial biopsy have improved the ability to characterize changes in the heart and facilitate the diagnosis of myocarditis (Friedrich et al., 2009). However, the molecular mechanisms underlying AM are not fully understood.

FM is the most severe form of AM, which is characterized by a rapidly progressing clinical course that results in serious hemodynamic compromise and even death (Gupta et al., 2008; Wang et al., 2019). Initially, FM might present heterogeneously with symptoms ranging from fever, lethargy, and myalgia to severe heart failure. Given the variability of symptoms, it may be difficult to differentiate FM from NFM at the disease onset. FM progresses to severely impaired LVEF more rapidly than NFM. Late gadolinium enhancement (LGE) cMRI shows that patients with FM exhibit a greater inflammatory involvement of the myocardium and a greater extent of edema and fibrosis than those with NFM (Ammirati et al., 2019; Sharma et al., 2019). However, the various pathophysiological mechanisms involved in the progression of FM and NFM remain elusive. Molecular investigations in the heart tissue, such as transcriptomics, could identify common and specific molecules or pathways in FM and NFM (Heidecker et al., 2011; Jin et al., 2017).

Non-coding RNAs (ncRNAs) are a class of non-coding ribonucleic acids that are critical contributors to various biological processes, among which long non-coding RNAs (lncRNAs) spanning more than 200 nucleotides in length are the major members (Batista and Chang, 2013). Our group and others have revealed that lncRNAs could regulate the occurrence and development of viral infection and that circulating non-coding RNAs might have a great value in the diagnosis of myocarditis (Chen et al., 2018; Nie et al., 2020; Zhang C et al., 2020). Meanwhile, the roles of lncRNAs in myocarditis have attracted increasing attention (Zhang et al., 2019; Xue et al., 2020; Zhang Y et al., 2020). For example, lncRNA MEG3 inhibits M2 macrophage polarization by activating TRAF6 *via* miRNA-223 downregulation in viral myocarditis (Xue et al., 2020). Altered lncRNA expression profiles were identified in the plasma by microarray analysis of children with FM (Liu et al., 2019). Nevertheless, the role of myocarditis-associated lncRNAs in the heart is still not fully understood.

In the current study, the expression profiles of lncRNAs in the heart of various CVB3-induced AM mice were assessed by RNA-seq. Several lncRNAs exhibited significantly altered expression levels, which were related to inflammatory responses in both FM and NFM. Furthermore, several lncRNAs showed distinct expression patterns in FM than in NFM, which indicated that lncRNAs might play important roles in AM, especially in FM.

## Materials And Methods

### Animals

This study was approved by the Institutional Animal Research Committee of Tongji Medical College. All animal experimental protocols complied with the Guide for the Care and Use of Laboratory Animals published by the National Institutes of Health. Male A/J (~6-week-old) mice were obtained from GemPharmatech (Nanjing, China). Male C57BL/6 (~6-week-old) mice, male BALB/c (~6-week-old) mice, and male C3H mice were purchased from Beijing Vital River Laboratory Animal Technology (Beijing, China). All four mice strains were randomly assigned to two groups: control (n = 10) and CVB3-treated (n = 15) groups. A/J mice and BALB/c mice were intraperitoneally injected with 10^4^ PFU CVB3, while C57BL/6 and C3H mice were intraperitoneally injected with 10^5^ PFU CVB3, as described previously (Huber et al., 2001; Althof et al., 2018). Phosphate-buffered saline (PBS) was used as the control. All animals were anesthetized with intraperitoneal injections of a mixture of xylazine (5 mg/kg) and ketamine (80 mg/kg). Echocardiography was performed on day 7 after virus injection, and the mice were sacrificed. Subsequently, the organs were collected and frozen in liquid nitrogen, followed by storage at −80°C.

### Cell Culture

HL-1 cells were cultured in Dulbecco’s modified Eagle’s medium (DMEM) supplemented with 10% fetal bovine serum (FBS) in a humidified atmosphere of 95% air and 5% CO_2_ at 37°C, as described previously (Fan et al., 2021). Cells were treated with CVB3 (1 × 10^4^ plaque forming unit) and collected 24 h later. Each experiment was repeated independently at least three times.

### LncRNA Sequencing Analysis

Heart tissue samples were collected from A/J, BALB/c, C57BL/6, and C3H mice. RNA isolation, quality control, library construction, and sequencing were performed by Personal Biotechnology Co. (Shanghai, China). TRIzol reagent (Invitrogen Life Technologies, CA, USA) was used to extract total RNA, and the concentration, quality, and integrity of RNAs were detected using a NanoDrop spectrophotometer (Thermo Scientific, CA, USA). Approximately 3 mg of RNA was used to generate RNA-Seq cDNA libraries. Sequencing libraries were then sequenced on a Hiseq X ten platform/NovaSeq 6000 (Illumina, San Diego, CA, USA). Cutadapt (v2.7) was used to filter the sequencing data and obtain clean reads for further analysis. The reference genome index was built using Bowtie2 (v2.4.1), and high-quality sequences were mapped to the reference genome using HISAT2 (v2.1.0). The resulting P-values were adjusted using Benjamini and Hochberg’s approach for controlling the false discovery rate. Genes with an adjusted P <0.05, and absolute LogFC >1 measured by DESeq2 were assigned as differentially expressed.

### Validation by Quantitative Real-Time PCR

Total RNA extracted from the heart tissue was transcribed into cDNA, and lncRNAs were quantified by qRT-PCR using Hieff qPCR SYBR Green Master Mix (Yeasen Biotech, Shanghai, China) on a 7900HT Fast Real-Time PCR system (Life Technologies, Carlsbad, CA, USA). qRT-PCR was performed using standard settings: 95°C for 2 min, 40 cycles of 95°C for 10 s, 60°C for 30 s, and 72°C for 15 s. LncRNA primers were designed by us and synthesized by AuGCT (Wuhan, China) (**Supplemental Table S1**). PCR reactions were performed in a total volume of 10 μl containing 1 μl of 100 ng/ml sample cDNA, 5 Ml of SYBR Green Master Mix, 0.5 μl of 5 mM forward primer, 0.5 μl of 5 mM reverse primer, and 3 μl of RNase/DNase-free water. Relative expression levels were calculated using the 2^-ΔΔCt^ relative quantification method, as previously described (Nie et al., 2020).

### Cardiac Function Detection in Mice

Echocardiography analysis was performed on day 7 after virus injection using a high-resolution imaging system with a 30-MHz high-frequency scanhead (VisualSonics Vevo770, VisualSonics, Toronto, Canada), as described previously (Nie et al., 2018).

### Histological Analysis

After echocardiography, the heart samples were collected, fixed with 4% paraformaldehyde, embedded in paraffin, and sectioned into 4 μm slices. Heart morphology was evaluated by hematoxylin and eosin (H&E) staining and measured using Image-Pro Plus Version 6.0 software (Media Cybernetics, Washington, USA). Six views of one H&E-stained section under a microscope were randomly collected and averaged as one data point. For each group, the results were analyzed using at least six groups of data.

### Functional Analysis of lncRNA Target Genes

Cis-target gene prediction was performed using the UCSC database (Haeussler et al., 2019). DAVID Bioinformatics Resources was employed for KEGG pathway analysis of the target genes. Statistical significance was set at P <0.05. MiRNAs targets of lncRNAs were predicted by miRnada and psRobot and analyzed by Cytoscape 3.7.

### Statistical Analysis

Data are presented as the mean ± standard error of the mean (SEM). Student’s t-test was used to compare the differences in normally distributed continuous values; Mann–Whitney test was used to evaluate the differences in non-normally distributed continuous values. Pearson or Spearman rank correlation was conducted to evaluate the relationships between candidate lncRNAs and clinical parameters according to the normal distribution. Statistical significance was set at P <0.05.

## Results

### Identification of Differentially Expressed lncRNAs in AM

**Figure 1:**
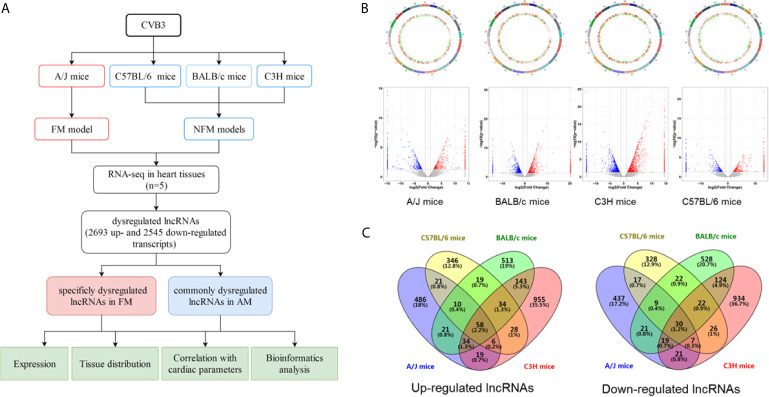
Identification of differentially expressed long non-coding RNAs (lncRNAs) in acute myocarditis (AM). **(A)** The flowchart of the present study. **(B)** Genetic map and volcano plot of the expression of lncRNAs in heart tissues from four mice strains (A/J mice for fulminant myocarditis; BALB/c mice, C3H mice and C57BL/6 mice for acute myocarditis). The red or blue plots represent up- or downregulated lncRNAs with P <0.05, whereas the black plots represent insignificant changes. **(C)** The Venn diagram shows the overlapping number of altered lncRNAs across the four myocarditis models.

C57BL/6, BALB/c, C3H, and A/J mice with CVB3-induced NFM and FM were established, as indicated by the inflammatory cell infiltration in the heart tissues accompanied by impaired cardiac function, respectively (**Supplemental Figures S1A, B**). RNA-seq was performed to evaluate the expression levels of the identified lncRNAs in the heart of the four models. A flowchart of the present study is shown in **Figure 1A**. A total of 655, 522, 832, and 1,277 lncRNAs were upregulated, while 561, 461, 774, and 1,182 transcripts were downregulated in A/J, C57BL/6, BALB/c, and C3H mice, respectively (**Figure 1B**). Among them, 58 upregulated lncRNAs and 30 downregulated lncRNAs were not only identified in A/J FM mice but also in three NFM models (**Figure 1C**). Among these commonly dysregulated lncRNAs, 27 lncRNAs (15 upregulated lncRNAs and 12 downregulated lncRNAs; FPKM >0 in at least two myocarditis models) were genetically structured or sequenced conservatively between mice and humans (**Supplemental Table S2**).

Compared with NFM mice models, CVB3-treated A/J mice usually exhibit more severe inflammatory infiltration and cardiac dysfunction, which are considered as a symptom of severe myocarditis [21, 22]. Here, we found that 486 and 437 lncRNAs were specifically up- and downregulated in CVB3-induced A/J murine myocarditis, respectively, as detected by RNA-seq (**Figure 1C**). The top 20 of these up- and downregulated lncRNAs are listed in **Supplemental Table S3**.

The results indicated that these commonly and FM-specific dysregulated lncRNAs might contribute to the pathogenesis of AM.

### Confirmation of Differentially Expressed lncRNAs in AM

**Figure 2:**
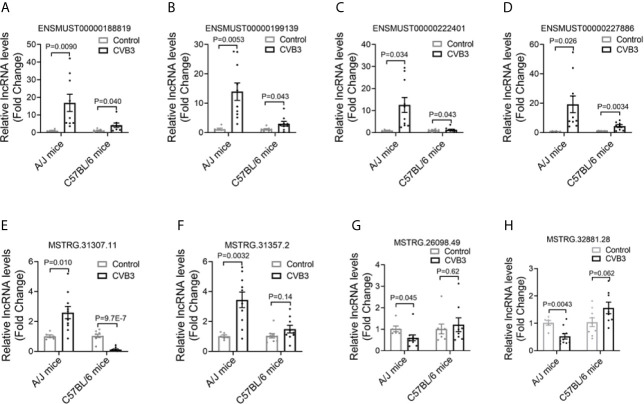
Confirmation of differentially expressed long non-coding RNAs (lncRNAs) in acute myocarditis (AM). The expression of **(A)** ENSMUST00000188819, **(B)** ENSMUST00000199139, **(C)** ENSMUST00000222401, **(D)** ENSMUST00000227886, **(E)** MSTRG.31307.11, **(F)** MSTRG.31357.2, **(G)** MSTRG.26098.49, and **(H)** MSTRG.32881.28 in the heart tissues from CVB3-treated mice was detected by quantitative real-time PCR (qRT-PCR).

To validate the changes in lncRNA expression detected by RNA-seq, six out of the 15 commonly upregulated lncRNAs were selected and assessed by qRT-PCR in CVB3-treated A/J and C57BL/6 mice. The expression levels of ENSMUST00000188819, ENSMUST00000199139, ENSMUST00000222401, and ENSMUST00000227886 were significantly increased, which was in line with the RNA-seq results (**Figures 2A–D**, **Supplemental Figures SA, B** **)**. Meanwhile, the expression levels of the top 10 FM-specific up- and downregulated lncRNAs were further validated by qRT-PCR. The expression levels of MSTRG.31307.11 and MSTRG.31357.2 were uniquely upregulated, while MSTRG.26098.49 and MSTRG.32881.28 levels were uniquely downregulated in FM mice, which were in line with the RNA-seq results (**Figures 2E–H**, **Supplemental Figure S2C–H**).

These results suggested that different lncRNAs might participate in FM and NFM.

#### Tissue Distributions of Differentially Expressed lncRNAs in AM

We first analyzed the expression pattern of ENSMUST 00000188819, ENSMUST00000199139, ENSMUST00000222401, and ENSMUST00000227886 in normal mouse tissues using the NONCODE database, which showed that they were mainly expressed in the spleen and thymus, with very low abundance in the heart (**Supplemental Figure S3A**).

**Figure 3:**
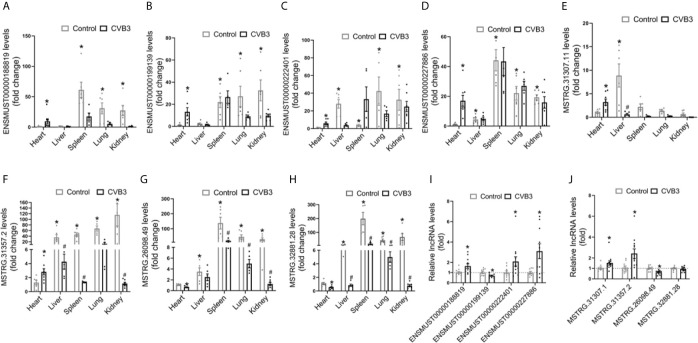
Tissue distributions of differentially expressed long non-coding RNAs (lncRNAs) in acute myocarditis (AM). The expression of **(A)** ENSMUST00000188819, **(B)** ENSMUST00000199139, **(C)** ENSMUST00000222401, **(D)** ENSMUST00000227886, **(E)** MSTRG.31307.11, **(F)** MSTRG.31357.2, **(G)** MSTRG.26098.49, and **(H)** MSTRG.32881 in organs from CVB3-treated A/J mice was detected by quantitative real-time PCR (qRT-PCR); *p < 0.05 *vs*. normal heart; ^#^p < 0.05 *vs*. control. The expression of commonly dysregulated lncRNAs **(I)** and FM-specific dysregulated lncRNAs **(J)** in HL-1 cardiomyocytes treated by CVB3; *p < 0.05 *vs*. control.

Then, we detected the expression levels of these four lncRNAs in various organs from CVB3-treated A/J mice by qRT-PCR. Although their expression levels in the heart were not the highest in normal mice, they increased significantly in the heart and spleen upon CVB3 treatment (**Figures 3A–D**). Additionally, the tissue distributions of FM-specific dysregulated lncRNAs were detected in A/J mice. MSTRG.31307.11 and MSTRG.31357.2 were only upregulated in the heart after CVB3 treatment (**Figures 3E, F** **)**, while the expression of MSTRG.26098.49 and MSTRG.32881.28 was significantly decreased (**Figures 3G, H** **)**.

Furthermore, we found that upon CVB3 activation, the expression of ENSMUST00000188819, ENSMUST00000222401, and ENSMUST00000227886 was elevated in cultured cardiomyocytes but that of ENSMUST00000199139 was decreased (**Figure 3I**). Similarly, CVB3 treatment also directly altered the expression of FM-specific dysregulated lncRNAs in cultured cardiomyocytes (**Figure 3J**).

These results indicated that these commonly upregulated lncRNAs, especially FM-specific upregulated lncRNAs, might play more vital roles in the heart than in other organs.

#### Correlation Between lncRNA Expression Levels and Cardiac Inflammatory Response

**Figure 4:**
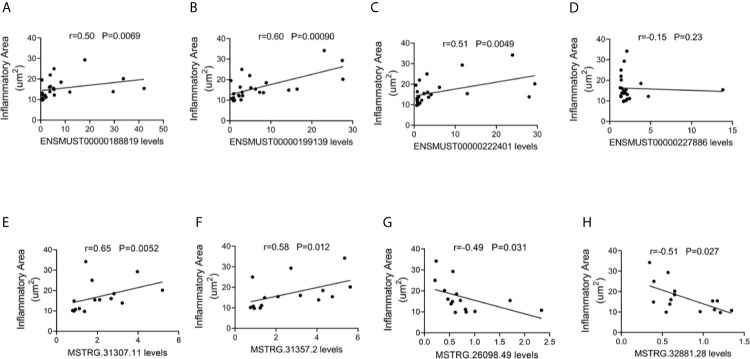
Correlation between long non-coding RNA (lncRNA) expression and cardiac inflammatory response. The correlation between the levels of **(A)** ENSMUST00000188819, **(B)** ENSMUST00000199139, **(C)** ENSMUST00000222401, **(D)** ENSMUST00000227886, **(E)** MSTRG.31307.11, **(F)** MSTRG.31357.2, **(G)** MSTRG.26098.49, and **(H)** MSTRG.32881.28 and the inflammatory area.

To explore the potential roles of lncRNAs in AM, the correlations between the four commonly dysregulated lncRNA levels and inflammatory infiltration in the heart of CVB3-treated A/J mice were evaluated. Among them, the expression levels of ENSMUST00000188819, ENSMUST00000199139, and ENSMUST00000222401 in the heart were positively correlated with cardiac inflammatory infiltration (**Figures 4A–D**).

For FM-specific dysregulated lncRNAs, MSTRG.31307.11, MSTRG.31357.2, MSTRG.26098.49, and MSTRG.32881.28 were correlated with cardiac inflammatory infiltration (**Figures 4E–H**).

These results suggested that these dysregulated lncRNAs, especially FM-specific dysregulated lncRNAs, might play important roles in immune responses during AM.

#### Correlation Between lncRNA Expression and Cardiac Function

**Figure 5:**
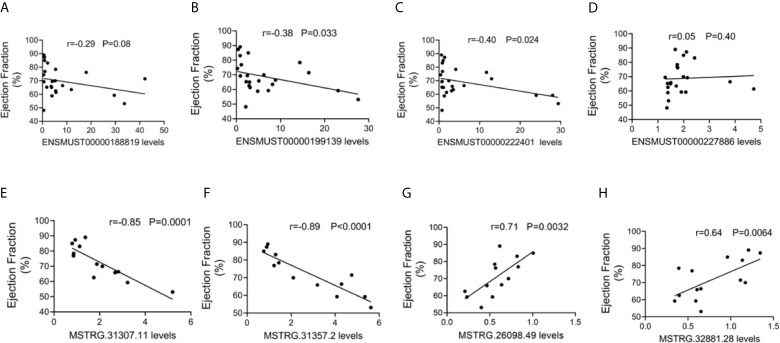
Correlation between long non-coding RNA (lncRNA) expression and cardiac function. The correlation between the levels of **(A)** ENSMUST00000188819, **(B)** ENSMUST00000199139, **(C)** ENSMUST00000222401, **(D)** ENSMUST00000227886, **(E)** MSTRG.31307.11, **(F)** MSTRG.31357.2, **(G)** MSTRG.26098.49, and **(H)** MSTRG.32881.28 and the ejection fraction (EF, %).

To further evaluate the potential function of the above-mentioned dysregulated lncRNAs, the associations between the expression levels of these lncRNAs and cardiac function were also analyzed. The expression levels of commonly dysregulated lncRNAs ENSMUST00000199139 and ENSMUST00000222401 were negatively correlated with LVEF in mice (**Figures 5A–D**). Moreover, FM-specific dysregulated lncRNAs were strongly correlated with LVEF in mice (**Figures 5E–H**).

These results suggest that FM-specific dysregulated lncRNAs might be more important in the regulation of cardiac function.

#### Bioinformatics Analyses of Dysregulated lncRNAs in AM

**Figure 6:**
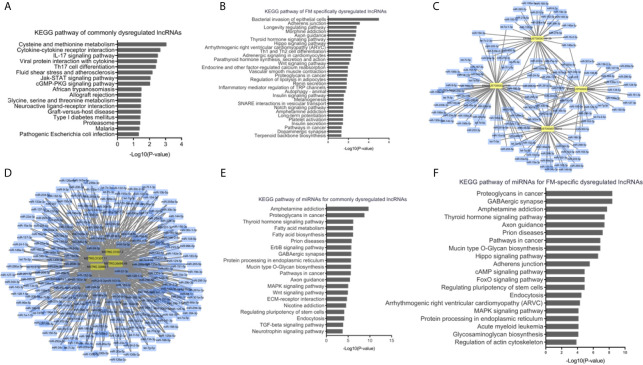
Bioinformatics analyses of dysregulated long non-coding RNAs (lncRNAs) in acute myocarditis (AM). **(A)** Kyoto Encyclopedia of Genes and Genomes **(KEGG)** pathway analyses for cis-target genes of commonly upregulated lncRNAs. **(B)** KEGG pathway analyses for cis-target genes of fulminant myocarditis (FM)-specific upregulated lncRNAs. **(C)** LncRNA-miRNAs networks for commonly upregulated lncRNAs. **(D)** LncRNA-miRNAs networks for FM-specific dysregulated lncRNAs. **(E)** KEGG pathway analyses for miRNAs targeted by commonly dysregulated lncRNAs. **(F)** KEGG pathway analyses for miRNAs targeted by FM-specific dysregulated lncRNAs.

To understand the dysregulated lncRNA-associated molecular mechanisms, bioinformatics analyses were performed. KEGG pathway analysis indicated that the cis-target genes of ENSMUST00000199139, ENSMUST00000188819, ENSMUST00000227886, and ENSMUST00000222401 were involved in multiple immune system pathways, such as the IL-17 signaling pathway, viral protein interaction with cytokines, and Th17 cell differentiation (**Figure 6A**), indicating the nature of this inflammatory disease in the heart. Cardiomyocyte damage and inflammation are the predominant histopathological changes associated with the progression of FM. KEGG analyses of the FM-specific dysregulated lncRNAs showed that pathways, such as bacterial invasion of epithelial cells, adherens junction, longevity regulating pathway, Hippo signaling pathway, and arrhythmogenic right ventricular cardiomyopathy (ARVC), were enhanced (**Figure 6B**).

Previous studies have indicated that lncRNAs could mediate biological processes by functioning as miRNA sponges. Therefore, interactions of the above lncRNAs with putative complementary miRNAs were assessed using Cytoscape 3.7. A series of miRNAs with critical roles in inflammation and cardiac function, such as miR-133a, miR-208a, miR-200a, and miR-34a, were identified as direct targets of commonly and FM-specific dysregulated lncRNAs (**Figures 6C, D** **)**. KEGG analyses of these targeted miRNAs indicated that pathways such as amphetamine addiction, thyroid hormone signaling pathway, prion diseases, and endocytosis were commonly enriched (**Figure 6E**). Nevertheless, pathways such as the Hippo signaling pathway, ARVC, and regulation of actin cytoskeleton were specifically involved in miRNAs targeted by FM-specific dysregulated lncRNAs (**Figure 6F**).

These results suggested that lncRNAs might regulate the pathophysiological mechanisms of AM through diverse signaling pathways.

## Discussion

In the current study, the expression profiles of lncRNAs in the heart were detected in CVB3-induced AM mouse models. Several lncRNAs were confirmed to have common or specific expression patterns in FM and NFM. Functional analyses of these dysregulated lncRNAs revealed the varied pathogenesis of FM and NFM.

Myocarditis is an inflammatory heart disease with variable clinical outcomes. There is firm evidence that enteroviruses account for many infections in cardiomyocytes. It is also thought that acute and chronic myocarditis might develop because of infection with adenoviruses, parvovirus B19 (PVB19), human herpesvirus 6, and Epstein–Barr virus (Cooper, 2009; Breinholt et al., 2010; Pollack et al., 2015). Pathogen-recognition receptors such as TLR-3 and TLR-4 recognize positive-sense single-stranded RNA viruses such as CVB3 (Garmaroudi et al., 2015). Mice deficient in TLR4 or TLR9 show attenuated myocarditis phenotypes accompanied by reduced production of inflammatory cytokines (Hollingsworth et al., 2004; Pagni et al., 2010). Animals lacking the damage-associated molecular patterns S100A8 and S100A9 showed reduced cardiac inflammation (Marinkovic et al., 2020; Muller et al., 2020). Upon infection, cardiac-resident cells, such as cardiomyocytes, endothelial cells, and fibroblasts, may contribute to acute inflammation by secreting cytokines such as IL-1β, IL-6, TNF-α, and IL-18. The inflammatory infiltrates in the EMB specimens are primarily composed of T lymphocytes and macrophages (Rivadeneyra et al., 2018). Various innate (NK cells and macrophages) and adaptive (T and B cells) immune cells infiltrate into tissues and contribute to tissue damage by secreting inflammatory cytokines (Epelman et al., 2015). Viral myocarditis presents with decreased ejection fraction and increased left ventricular end-diastolic diameter and is usually associated with cardiomyocyte loss (Sagar et al., 2012). Canonical activation of the inflammasome is critical to promote caspase-1-dependent maturation of the proinflammatory cytokines IL-1β and IL-18, as well as to induce cell death in response to pathogens (Rathinam and Fitzgerald, 2016; Toldo et al., 2018). The classical inflammasome complex consists of a cytosolic sensor, an adaptor protein ASC, and an effector caspase, pro-caspase-1. Activated pro-caspase-1 promotes the cleavage of pro-IL-1β and pro-IL-18 and the generation of biologically mature active cytokines. In addition to the canonical mode of inflammasome activation, additional caspases, such as caspase-8 or caspase-11 (caspase 4/5 in humans), also contribute to inflammasome-dependent control of IL-1β/IL18 processing and pyroptotic cell death (Rathinam and Fitzgerald, 2016).

Viral infections are the most common cause of myocarditis, which is usually caused by viruses such as enteroviruses and adeno viruses (Cooper, 2009). Modern advances in PCR technology have enabled the detection of human herpesvirus 6 and parvovirus B19 in patients with AM (Breinholt et al., 2010; Pollack et al., 2015). Coxsackieviruses belong to the Enterovirus genus within the Picornaviridae family, comprising of six Coxsackieviruses group B (CVB). Among the six serotypes of CVB, only CVB1, CVB3, and CVB5 are notably cardiotropic (Gauntt et al., 1983; Garmaroudi et al., 2015). Group B Coxsackieviruses frequently cause cardiac inflammation in humans. In murine models of CVB3-induced myocarditis, a wide spectrum of histopathology was observed, which could mimic the diverse processes in diseased patients (Garmaroudi et al., 2015). CVB3 induced myocarditis in susceptible strains of mice, such as A/J and BALB/c mice, has provided insights into the pathogenesis of viral myocarditis, as it shares many biological parameters of CVB3-induced disease in humans (Gauntt and Huber, 2003; Fairweather et al., 2012). In addition to viral infections, *Trypanosoma cruzi* and bacterial infections still contribute to the global burden of acute myocarditis in the developing world (Sagar et al., 2012). The lncRNA expression pattern and their potential differentiation function in these factors mediated by FM and NFM require further study.

FM is the most severe form of myocarditis. Patients with FM present with acute, severe heart failure, cardiogenic shock, and even death. Moreover, patients with FM also exhibit a greater inflammatory involvement of the myocardium and more severely impaired LVEF than those with NFM (Ammirati et al., 2019). Cardiomyocyte damage and inflammation are the predominant histopathological changes in the progression of FM (Shirani et al., 1993; Maisch et al., 2014). Here, RNA-seq was performed in the heart tissue from CVB3-induced mouse models to differentiate FM from NFM. Several dysregulated lncRNAs were detected with vital roles in the pathogenesis of FM. Longevity regulating and Hippo signaling pathways are vital contributors to cell growth and death (Zheng and Pan, 2019). FM-specific dysregulated lncRNAs might be core mediators of the longevity regulating pathway and cell growth, which play more important roles in cardiac function than the commonly dysregulated lncRNAs during AM.

Non-coding RNAs (ncRNAs) are a class of transcriptional RNAs with no protein-coding potential, mainly comprising lncRNAs, circRNAs, and miRNAs. ncRNAs are known to play vital roles in viral infection and inflammatory responses (Chen et al., 2018). Altered expression of lncRNAs in response to viral infection in eukaryotes has been reported in recent studies (Peng et al., 2010; Zhang et al., 2016; Liu et al., 2019). LncRNA HIF1A-AS1 levels were significantly increased in CVB3-induced myocardium and cardiomyocytes, and silencing of HIF1A-AS1 reduced the release of pro-inflammatory cytokines and alleviated the rate of late apoptosis and ROS production by targeting miR-138 (Cao et al., 2020). Downregulation of lncRNA-MEG3 leads to the inhibition of inflammation and induces M2 macrophage polarization *via* the miR-223/TRAF6/NF-κB axis in CVB3-induced viral myocarditis (Xue et al., 2020). LncRNA AK085865 promotes macrophage M2 polarization by regulating the ILF2-ILF3 complex in CVB3-induced viral myocarditis (Zhang Y et al., 2020). In addition to the host cell generating diverse lncRNAs, the viruses themselves express ncRNAs to mediate cellular antiviral activity and immune response. Human adenovirus-encoded VA RNAs bind dicer and function as competitive substrate-suppressing dicer to inhibit RNAi. VA RNA also binds and consequently blocks PKR activity and inhibits the activation of eIF-2a and viral mRNA translation (Svensson and Akusjärvi, 1984; Smith et al., 1994). Although much effort has been made to understand the effect of lncRNAs in myocarditis, the features of lncRNAs in different subtypes of acute myocarditis remain unknown. In this study, we identified and confirmed the variably and commonly expressed lncRNAs and preliminarily disclosed their potential roles in FM and NFM. However, the exact function of lncRNAs in myocarditis requires further study.

LncRNA ENSMUST00000222401, termed DIO3OS, was commonly upregulated in four mouse myocarditis models. Previously, the function of DIO3OS has been mainly studied in cancers. DIO3OS levels were considerably increased in the thyroid cancer tissue samples. Knocking down DIO3OS within thyroid carcinoma cells suppressed cancer cell viability, cell invasion, and migration *via* regulation of the let-7d/NF-κB2 axis (Wang et al., 2021). Another study showed that DIO3OS exhibited oncogenic properties by stimulating pancreatic cancer cell proliferation and invasion and promoting cancer growth by binding to miRNA-122 (Cui et al., 2019). Conversely, DIO3OS levels were lower in hepatocellular carcinoma tissues, and upregulation of DIO3OS repressed malignant biological behavior by sponging miR-328 (Wang et al., 2020). Other studies have also shown that the expression levels of DIO3OS were significantly lower in patients with Crohn’s disease and ulcerative colitis than those in healthy controls. The area under the receiver operating characteristic (ROC) curve between DIO3OS expression in patients with Crohn’s disease or ulcerative colitis and the healthy controls was 0.794 and 0.653, respectively, which suggests that DIO3OS has potential diagnostic value for detecting inflammatory bowel disease (Wang et al., 2018). This study suggested that lncRNA DIO3OS is related to inflammation, but the role of DIO3OS in myocarditis requires further study.

Interestingly, the expression level of ENSMUST00000199139 was increased in heart tissues but decreased in cultured cardiomyocytes upon CVB3 treatment. A similar finding was also reported in our previous study. MiR-320 levels were significantly elevated in cardiomyocytes but were decreased in cardiac fibroblasts, thereby leading to a slight increase in miR-320 expression in the global heart tissue of TAC mice (Zhang et al., 2021). Moreover, another study showed a decreased expression trend in SARS-CoV-2-infected human induced pluripotent stem cell (iPSC)-derived cardiomyocytes, as detected by RNA-seq (Sharma et al., 2020). These results suggested that ENSMUST00000199139 might be expressed by multiple cell types with different changes upon CVB3 infection. In fact, we have explored ENSMUST00000199139 expression in other cell types of the heart in A/J mice by RNA-FISH. The results showed that ENSMUST00000199139 expression was mainly increased in macrophages upon CVB3 infection (data not shown), and its function requires further investigation.

In this study, we reported altered expression profiles of lncRNAs and their correlation with cardiac function and inflammatory response in myocarditis in detail; however, there are still limitations to this study. Further in-depth studies are needed to demonstrate the exact biological function of lncRNAs in myocarditis and to investigate the diagnostic value of differential lncRNAs in plasma. Noteworthily, results of lncRNA expression levels might vary across the source material, platforms, and sample sizes, requiring careful and critical evaluation when interpreting findings and comparing results from different groups.

## Data Availability Statement

The datasets presented in this study can be found in online repositories. The names of the repository/repositories and accession number(s) can be found below: The ArrayExpress database at EMBL-EBI (www.ebi.ac.uk/arrayexpress) under accession number E-MTAB-10823.

## Ethics Statement

This study was approved by the Institutional Animal Research Committee of Tongji Medical College. All animal experimental protocols complied with the Guide for the Care and Use of Laboratory Animals published by the NIH and Animal Research.

## Author Contributions

XN and HL designed the study, conducted the experiments, analyzed and interpreted the data, and drafted the paper. JW, YC, JF, and BD contributed to the data acquisition. DWW and CC designed the study and drafted the manuscript. All authors contributed to the article and approved the submitted version.

## Funding

This work was supported by grants from the National Natural Science Foundation of China [91839302, 81630010, and 81790624 to DW], the Natural Science Foundation of Hubei Province [2020CFA016 to CC], and the Tongji Hospital Clinical Research Flagship Program [2019CR207 to DW]. No funding bodies had any role in the study design, data collection and analysis, decision to publish, or preparation of the manuscript. All authors were fully aware of and approved the submission of this manuscript.

## Conflict of Interest

The authors declare that the research was conducted in the absence of any commercial or financial relationships that could be construed as a potential conflict of interest.

## Publisher’s Note

All claims expressed in this article are solely those of the authors and do not necessarily represent those of their affiliated organizations, or those of the publisher, the editors and the reviewers. Any product that may be evaluated in this article, or claim that may be made by its manufacturer, is not guaranteed or endorsed by the publisher.

## References

[B1] Althof N. Goetzke C. C. Kespohl M. Voss K. Heuser A. Pinkert S.. (2018). The Immunoproteasome-Specific Inhibitor ONX 0914 Reverses Susceptibility to Acute Viral Myocarditis. EMBO Mol. Med. 10 (2), 200–218. 10.15252/emmm.201708089 29295868 PMC5801517

[B2] Ammirati E. Veronese G. Brambatti M. Merlo M. Cipriani M. Potena L.. (2019). Fulminant Versus Acute Nonfulminant Myocarditis in Patients With Left Ventricular Systolic Dysfunction. J. Am. Coll. Cardiol. 74 (3), 299–311. 10.1016/j.jacc.2019.04.063 31319912

[B3] Batista P. J. Chang H. Y. (2013). Long Noncoding RNAs: Cellular Address Codes in Development and Disease. Cell 152 (6), 1298–1307. 10.1016/j.cell.2013.02.012 23498938 PMC3651923

[B4] Breinholt J. P. Moulik M. Dreyer W. J. Denfield S. W. Kim J. J. Jefferies J. L.. (2010). Viral Epidemiologic Shift in Inflammatory Heart Disease: The Increasing Involvement of Parvovirus B19 in the Myocardium of Pediatric Cardiac Transplant Patients. J. Heart Lung Transplant. 29 (7), 739–746. 10.1016/j.healun.2010.03.003 20456978 PMC2902647

[B5] Caforio A. L. Pankuweit S. Arbustini E. Basso C. Gimeno-Blanes J. Felix S. B.. (2013). Current State of Knowledge on Aetiology, Diagnosis, Management, and Therapy of Myocarditis: A Position Statement of the European Society of Cardiology Working Group on Myocardial and Pericardial Diseases. Eur. Heart J. 34 (33), 2636–2648, 2648a-2648d. 10.1093/eurheartj/eht210 23824828

[B6] Cao H. Yang B. Zhao Y. Deng X. Shen X. (2020). The Pro-Apoptosis and Pro-Inflammation Role of LncRNA HIF1A-AS1 in Coxsackievirus B3-Induced Myocarditis via Targeting miR-138. Cardiovasc. Diagn. Ther. 10 (5), 1245–1255. 10.21037/cdt-20-545 33224748 PMC7666947

[B7] Chen L. Zhou Y. Li H. (2018). LncRNA, miRNA and lncRNA-miRNA Interaction in Viral Infection. Virus Res. 257, 25–32. 10.1016/j.virusres.2018.08.018 30165080

[B8] Cooper L. T. Jr. (2009). Myocarditis. N. Engl. J. Med. 360 (15), 1526–1538. 10.1056/NEJMra0800028 19357408 PMC5814110

[B9] Cui K. Jin S. Du Y. Yu J. Feng H. Fan Q.. (2019). Long Noncoding RNA DIO3OS Interacts With miR-122 to Promote Proliferation and Invasion of Pancreatic Cancer Cells Through Upregulating ALDOA. Cancer Cell Int. 19, 202. 10.1186/s12935-019-0922-y 31384177 PMC6668142

[B10] Epelman S. Liu P. P. Mann D. L. (2015). Role of Innate and Adaptive Immune Mechanisms in Cardiac Injury and Repair. Nat. Rev. Immunol. 15 (2), 117–129. 10.1038/nri3800 25614321 PMC4669103

[B11] Fairweather D. Stafford K. A. Sung Y. K. (2012). Update on Coxsackievirus B3 Myocarditis. Curr. Opin. Rheumatol. 24 (4), 401–407. 10.1097/BOR.0b013e328353372d 22488075 PMC4536812

[B12] Fan J. Li H. Xie R. Zhang X. Nie X. Shi X.. (2021). LncRNA ZNF593-AS Alleviates Contractile Dysfunction in Dilated Cardiomyopathy. Circ. Res. 128 (11), 1708–1723. 10.1161/CIRCRESAHA.120.318437 33550812

[B13] Friedrich M. G. Sechtem U. Schulz-Menger J. Holmvang G. Alakija P. Cooper L. T.. (2009). Cardiovascular Magnetic Resonance in Myocarditis: A JACC White Paper. J. Am. Coll. Cardiol. 53 (17), 1475–1487. 10.1016/j.jacc.2009.02.007 19389557 PMC2743893

[B14] Fung G. Luo H. Qiu Y. Yang D. McManus B. (2016). Myocarditis. Circ. Res. 118 (3), 496–514. 10.1161/CIRCRESAHA.115.306573 26846643

[B15] Garmaroudi F. S. Marchant D. Hendry R. Luo H. Yang D. Ye X.. (2015). Coxsackievirus B3 Replication and Pathogenesis. Future Microbiol. 10 (4), 629–653. 10.2217/fmb.15.5 25865198

[B16] Gauntt C. Huber S. (2003). Coxsackievirus Experimental Heart Diseases. Front. Biosci. 8, e23–e35. 10.2741/928 12456330

[B17] Gauntt C. J. Paque R. E. Trousdale M. D. Gudvangen R. J. Barr D. T. Lipotich G. J.. (1983). Temperature-Sensitive Mutant of Coxsackievirus B3 Establishes Resistance in Neonatal Mice That Protects Them During Adolescence Against Coxsackievirus B3-Induced Myocarditis. Infect. Immun. 39 (2), 851–864. 10.1128/iai.39.2.851-864.1983 6299950 PMC348027

[B18] Gupta S. Markham D. W. Drazner M. H. Mammen P. P. (2008). Fulminant Myocarditis. Nat. Clin. Pract. Cardiovasc. Med. 5 (11), 693–706. 10.1038/ncpcardio1331 18797433

[B19] Haeussler M. Zweig A. S. Tyner C. Speir M. L. Rosenbloom K. R. Raney B. J.. (2019). The UCSC Genome Browser Database: 2019 Update. Nucleic Acids Res. 47 (D1), D853–d858. 10.1093/nar/gky1095 30407534 PMC6323953

[B20] Heidecker B. Kittleson M. M. Kasper E. K. Wittstein I. S. Champion H. C. Russell S. D.. (2011). Transcriptomic Biomarkers for the Accurate Diagnosis of Myocarditis. Circulation 123 (11), 1174–1184. 10.1161/CIRCULATIONAHA.110.002857 21382894 PMC3408077

[B21] Hollingsworth J. W. 2nd Cook D. N. Brass D. M. Walker J. K. Morgan D. L. Foster W. M.. (2004). The Role of Toll-Like Receptor 4 in Environmental Airway Injury in Mice. Am. J. Respir. Crit. Care Med. 170 (2), 126–132. 10.1164/rccm.200311-1499OC 15020293

[B22] Huber S. A. Graveline D. Born W. K. O’Brien R. L. (2001). Cytokine Production by Vgamma(+)-T-Cell Subsets Is an Important Factor Determining CD4(+)-Th-Cell Phenotype and Susceptibility of BALB/c Mice to Coxsackievirus B3-Induced Myocarditis. J. Virol. 75 (13), 5860–5869. 10.1128/jvi.75.13.5860-5869.2001 11390587 PMC114301

[B23] Jin J. Li R. Jiang C. Zhang R. Ge X. Liang F.. (2017). Transcriptome Analysis Reveals Dynamic Changes in Coxsackievirus A16 Infected HEK 293T Cells. BMC Genomics 18 (Suppl 1), 933. 10.1186/s12864-016-3253-6 28198671 PMC5310284

[B24] Liu Q. Kong Y. Han B. Jiang D. Jia H. Zhang L. (2019). Long Non-Coding RNA Expression Profile and Functional Analysis in Children With Acute Fulminant Myocarditis. Front. Pediatr. 7:283. 10.3389/fped.2019.00283 31355167 PMC6637775

[B25] Maisch B. Ruppert V. Pankuweit S. (2014). Management of Fulminant Myocarditis: A Diagnosis in Search of Its Etiology But With Therapeutic Options. Curr. Heart Fail. Rep. 11 (2), 166–177. 10.1007/s11897-014-0196-6 24723087

[B26] Marinkovic G. Koenis D. S. de Camp L. Jablonowski R. Graber N. de Waard V.. (2020). S100A9 Links Inflammation and Repair in Myocardial Infarction. Circ. Res. 127 (5), 664–676. 10.1161/CIRCRESAHA.120.315865 32434457

[B27] Muller I. Vogl T. Kuhl U. Krannich A. Banks A. Trippel T.. (2020). Serum Alarmin S100A8/S100A9 Levels and Its Potential Role as Biomarker in Myocarditis. ESC Heart Fail 7 (4), 1442–1451. 10.1002/ehf2.12760 32462801 PMC7373886

[B28] Nie X. Fan J. Li H. Yin Z. Zhao Y. Dai B.. (2018). miR-217 Promotes Cardiac Hypertrophy and Dysfunction by Targeting PTEN. Mol. Ther. Nucleic Acids 12, 254–266. 10.1016/j.omtn.2018.05.013 30195764 PMC6005806

[B29] Nie X. He M. Wang J. Chen P. Wang F. Lai J.. (2020). Circulating miR-4763-3p Is a Novel Potential Biomarker Candidate for Human Adult Fulminant Myocarditis. Mol. Ther. - Methods Clin. Dev. 17, 1079–1087. 10.1016/j.omtm.2020.05.005 32478123 PMC7248292

[B30] Pagni P. P. Traub S. Demaria O. Chasson L. Alexopoulou L. (2010). Contribution of TLR7 and TLR9 Signaling to the Susceptibility of MyD88-Deficient Mice to Myocarditis. Autoimmunity 43 (4), 275–287. 10.3109/08916930903509056 20187710

[B31] Peng X. Gralinski L. Armour C. D. Ferris M. T. Thomas M. J. Proll S.. (2010). Unique Signatures of Long Noncoding RNA Expression in Response to Virus Infection and Altered Innate Immune Signaling. mBio 1 (5), e00206-10. 10.1128/mBio.00206-10 20978541 PMC2962437

[B32] Pollack A. Kontorovich A. R. Fuster V. Dec G. W. (2015). Viral Myocarditis–Diagnosis, Treatment Options, and Current Controversies. Nat. Rev. Cardiol. 12 (11), 670–680. 10.1038/nrcardio.2015.108 26194549

[B33] Rathinam V. A. Fitzgerald K. A. (2016). Inflammasome Complexes: Emerging Mechanisms and Effector Functions. Cell 165 (4), 792–800. 10.1016/j.cell.2016.03.046 27153493 PMC5503689

[B34] Rivadeneyra L. Charo N. Kviatcovsky D. de la Barrera S. Gomez R. M. Schattner M. (2018). Role of Neutrophils in CVB3 Infection and Viral Myocarditis. J. Mol. Cell Cardiol. 125, 149–161. 10.1016/j.yjmcc.2018.08.029 30393107

[B35] Sagar S. Liu P. P. Cooper L. T. (2012). Myocarditis. Lancet 379 (9817), 738–747. 10.1016/s0140-6736(11)60648-x 22185868 PMC5814111

[B36] Sharma A. Garcia G. Jr. Wang Y. Plummer J. T. Morizono K. Arumugaswami V.. (2020). Human iPSC-Derived Cardiomyocytes Are Susceptible to SARS-CoV-2 Infection. Cell Rep. Med. 1 (4):100052. 10.1016/j.xcrm.2020.100052 32835305 PMC7323681

[B37] Sharma A. N. Stultz J. R. Bellamkonda N. Amsterdam E. A. (2019). Fulminant Myocarditis: Epidemiology, Pathogenesis, Diagnosis, and Management. Am. J. Cardiol. 124 (12), 1954–1960. 10.1016/j.amjcard.2019.09.017 31679645

[B38] Shirani J. Freant L. J. Roberts W. C. (1993). Gross and Semiquantitative Histologic Findings in Mononuclear Cell Myocarditis Causing Sudden Death, and Implications for Endomyocardial Biopsy. Am. J. Cardiol. 72 (12), 952–957. 10.1016/0002-9149(93)91113-V 8213554

[B39] Smith P. P. Dheer S. Davis A. Hung P. P. Lee S. G. (1994). Isolation and Characterization of Adenovirus-Associated VA RNAs of Human Adenovirus Type 7. Gene 142 (2), 309–310. 10.1016/0378-1119(94)90281-x 8194770

[B40] Svensson C. Akusjärvi G. (1984). Adenovirus VA RNAI: A Positive Regulator of mRNA Translation. Mol. Cell. Biol. 4 (4), 736–742. 10.1128/mcb.4.4.736 6201722 PMC368790

[B41] Toldo S. Mauro A. G. Cutter Z. Abbate A. (2018). Inflammasome, Pyroptosis, and Cytokines in Myocardial Ischemia-Reperfusion Injury. Am. J. Physiol. Heart Circ. Physiol. 315 (6), H1553–H1568. 10.1152/ajpheart.00158.2018 30168729 PMC6336966

[B42] Wang S. Hou Y. Chen W. Wang J. Xie W. Zhang X.. (2018). KIF9AS1, LINC01272 and DIO3OS lncRNAs as Novel Biomarkers for Inflammatory Bowel Disease. Mol. Med. Rep. 17 (2), 2195–2202. 10.3892/mmr.2017.8118 29207070 PMC5783463

[B43] Wang D. Li S. Jiang J. Yan J. Zhao C. Wang Y.. (2019). Chinese Society of Cardiology Expert Consensus Statement on the Diagnosis and Treatment of Adult Fulminant Myocarditis. Sci. China Life Sci. 62 (2), 187–202. 10.1007/s11427-018-9385-3 30519877 PMC7102358

[B44] Wang M. Li J. Zuo Z. Ren C. Tang T. Long C.. (2021). Long non-Coding RNA DIO3OS/let-7d/NF-Kappab2 Axis Regulates Cells Proliferation and Metastasis of Thyroid Cancer Cells. J. Cell Commun. Signal 15 (2), 237–250. 10.1007/s12079-020-00589-w 33058043 PMC7990978

[B45] Wang Z. Song L. Ye Y. Li W. (2020). Long Noncoding RNA DIO3OS Hinders Cell Malignant Behaviors of Hepatocellular Carcinoma Cells Through the microRNA-328/Hhip Axis. Cancer Manag. Res. 12, 3903–3914. 10.2147/CMAR.S245990 32547226 PMC7259459

[B46] Xue Y. L. Zhang S. X. Zheng C. F. Li Y. F. Zhang L. H. Su Q. Y.. (2020). Long non-Coding RNA MEG3 Inhibits M2 Macrophage Polarization by Activating TRAF6 *via* microRNA-223 Down-Regulation in Viral Myocarditis. J. Cell Mol. Med. 24 (21), 12341–12354. 10.1111/jcmm.15720 33047847 PMC7686963

[B47] Zhang Q. Lai M. M. Lou Y. Y. Guo B. H. Wang H. Y. Zheng X. Q. (2016). Transcriptome Altered by Latent Human Cytomegalovirus Infection on THP-1 Cells Using RNA-Seq. Gene 594 (1), 144–150. 10.1016/j.gene.2016.09.014 27623506 PMC7126988

[B48] Zhang Y. Li X. Wang C. Zhang M. Yang H. Lv K. (2020). lncRNA AK085865 Promotes Macrophage M2 Polarization in CVB3-Induced VM by Regulating ILF2-ILF3 Complex-Mediated miRNA-192 Biogenesis. Mol. Ther. Nucleic Acids 21, 441–451. 10.1016/j.omtn.2020.06.017 32668391 PMC7358220

[B49] Zhang C. Xiong Y. Zeng L. Peng Z. Liu Z. Zhan H.. (2020). The Role of Non-Coding RNAs in Viral Myocarditis. Front. Cell Infect. Microbiol. 10:312. 10.3389/fcimb.2020.00312 32754448 PMC7343704

[B50] Zhang X. Yuan S. Li H. Zhan J. Wang F. Fan J.. (2021). The Double Face of miR-320: Cardiomyocytes-Derived miR-320 Deteriorated While Fibroblasts-Derived miR-320 Protected Against Heart Failure Induced by Transverse Aortic Constriction. Signal Transduct. Target Ther. 6 (1), 69. 10.1038/s41392-020-00445-8 33597502 PMC7890065

[B51] Zhang M. Zheng Y. Sun Y. Li S. Chen L. Jin X.. (2019). Knockdown of NEAT1 Induces Tolerogenic Phenotype in Dendritic Cells by Inhibiting Activation of NLRP3 Inflammasome. Theranostics 9 (12), 3425–3442. 10.7150/thno.33178 31281488 PMC6587165

[B52] Zheng Y. Pan D. (2019). The Hippo Signaling Pathway in Development and Disease. Dev. Cell 50 (3), 264–282. 10.1016/j.devcel.2019.06.003 31386861 PMC6748048

