# Supplementary figures and images for: Expression Profiles and Potential Functions of Long Non-Coding RNAs in the Heart of Mice With Coxsackie B3 Virus-Induced Myocarditis
Source: Front Cell Infect Microbiol. 2021 Aug 24;11:704919. doi: 10.3389/fcimb.2021.704919 (PMC8423026; doi:10.3389/fcimb.2021.704919)

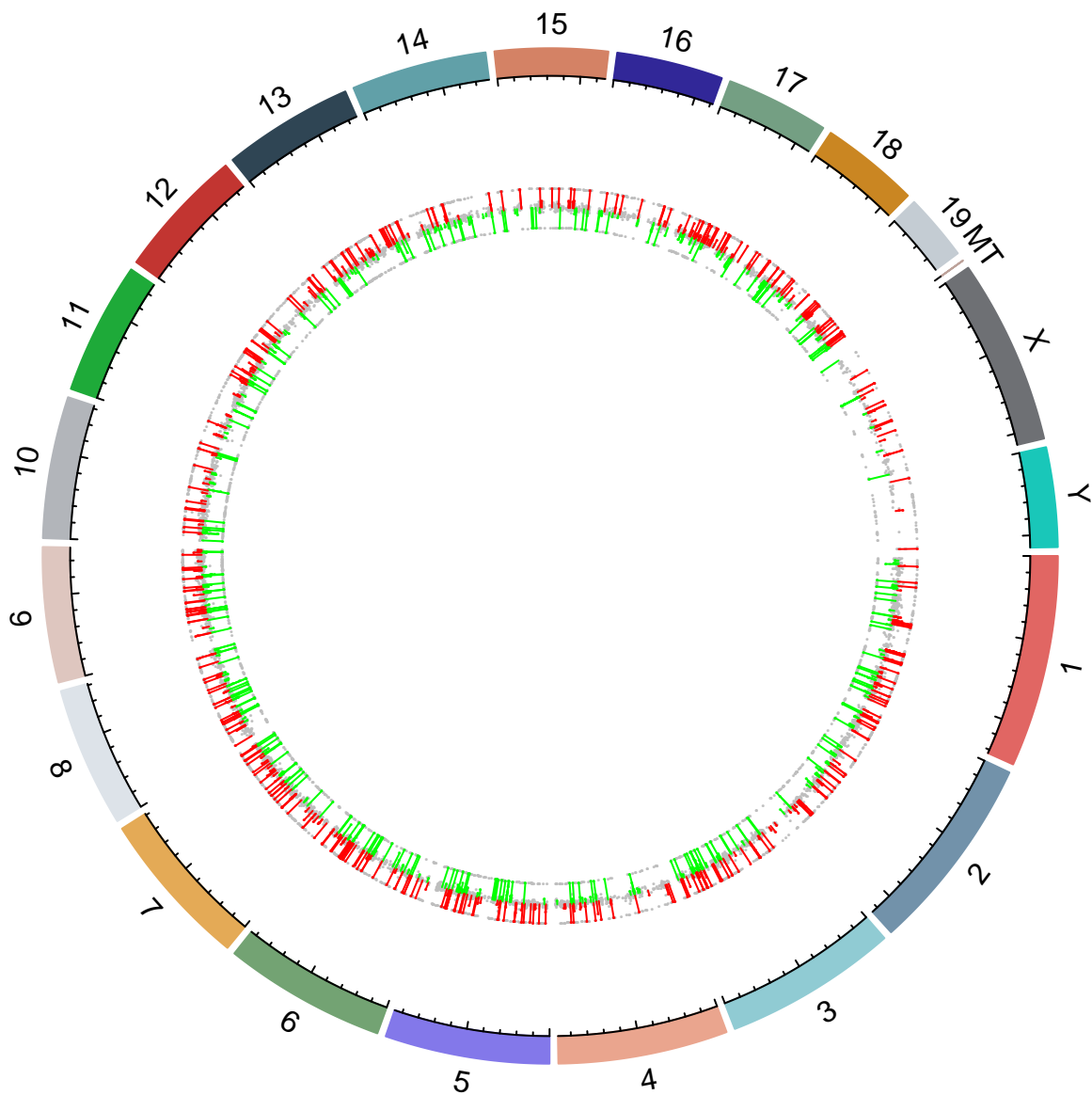

Supplement: Supplementary file 1 [file DataSheet_1.zip › Figure 1(raw data)/Figure 1B-1C/RNA-seq in A_J mice/genomeCircos-A_J mice/genomeCircos.pdf]

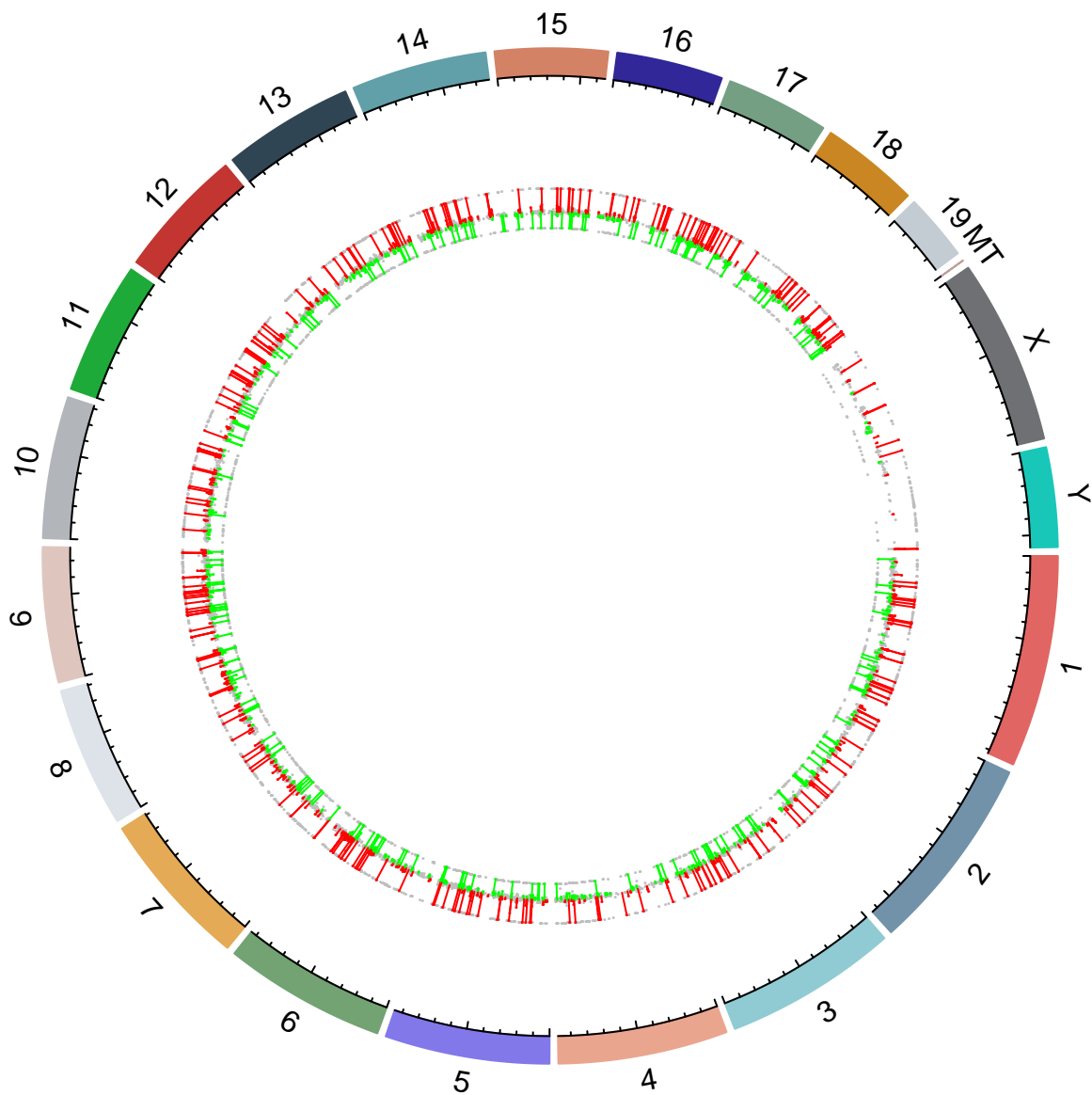

Supplement: Supplementary file 1 [file DataSheet_1.zip › Figure 1(raw data)/Figure 1B-1C/RNA-seq in BALBc mice/genomeCircos/genomeCircos.pdf]

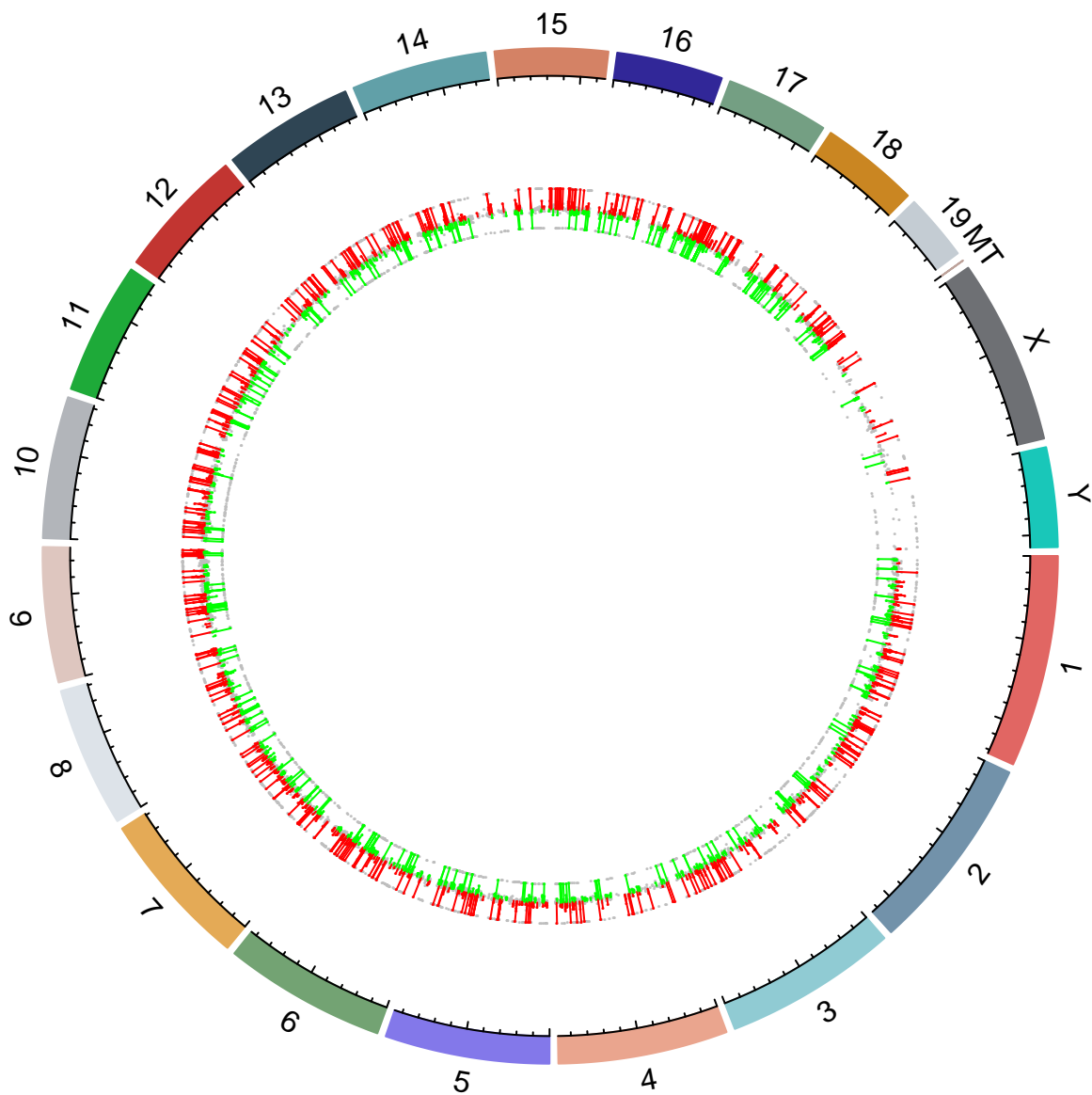

Supplement: Supplementary file 1 [file DataSheet_1.zip › Figure 1(raw data)/Figure 1B-1C/RNA-seq in C3H mice/genomeCircos/genomeCircos.pdf]

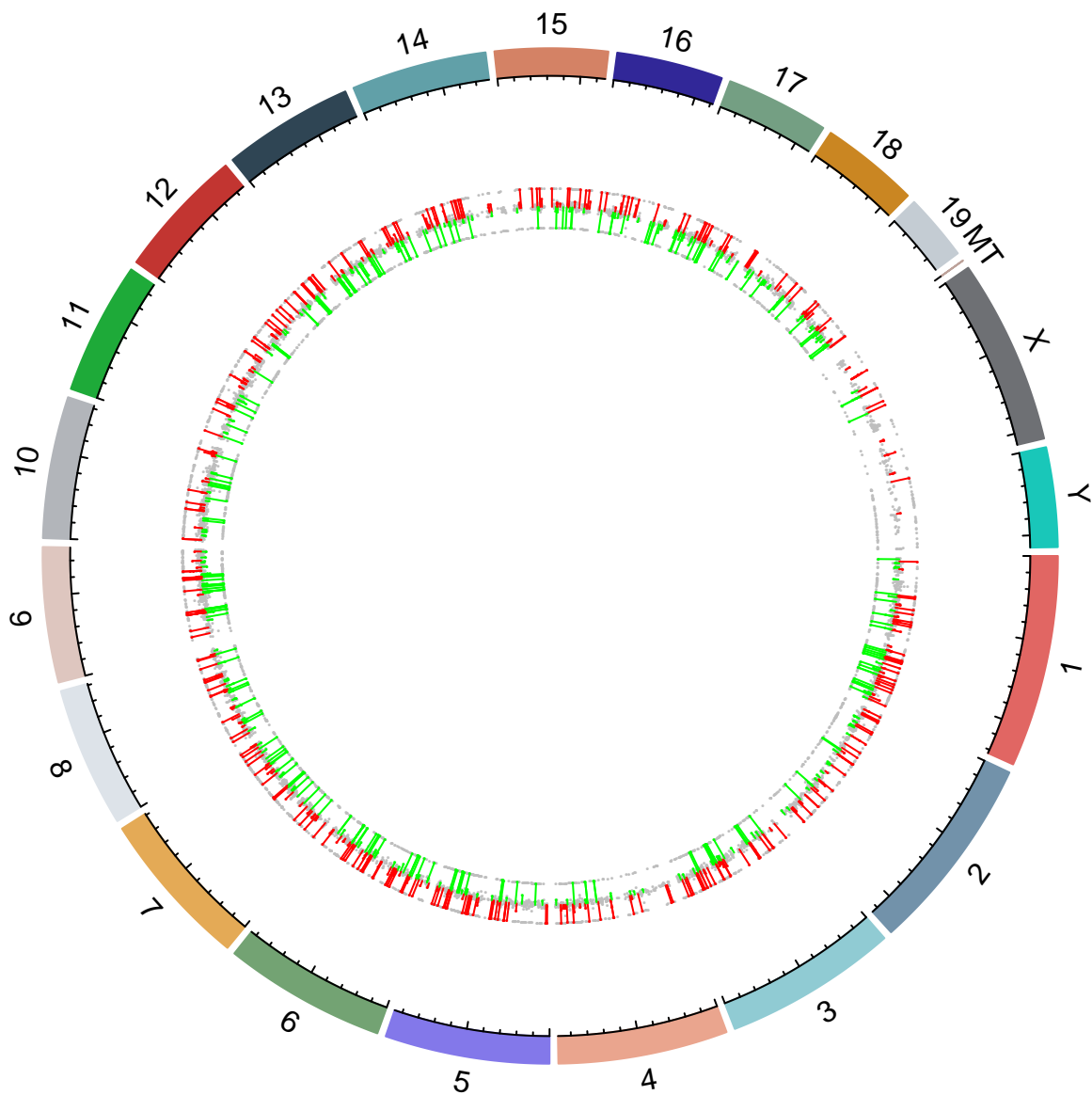

Supplement: Supplementary file 1 [file DataSheet_1.zip › Figure 1(raw data)/Figure 1B-1C/RNA-seq in C57BL_6 mice/genomeCircos/genomeCircos.pdf]
