# Supplementary material for: Expression Profiles and Potential Functions of Long Non-Coding RNAs in the Heart of Mice With Coxsackie B3 Virus-Induced Myocarditis
Source: Front Cell Infect Microbiol. 2021 Aug 24;11:704919. doi: 10.3389/fcimb.2021.704919 (PMC8423026; doi:10.3389/fcimb.2021.704919)
Supplement: Supplementary file 7 [file DataSheet_7.zip › Figure 6(raw data)/Figure 6A-6B and 6E-6F.docx]

Note:

Functions of lncRNAs and miRNAs were analyzed through database of KOBAS（<http://kobas.cbi.pku.edu.cn/genelist/）and> mirPathv.3 (<http://snf-515788.vm.okeanos.grnet.gr/>) respectively.
