# Supplementary material for: Expression Profiles and Potential Functions of Long Non-Coding RNAs in the Heart of Mice With Coxsackie B3 Virus-Induced Myocarditis
Source: Front Cell Infect Microbiol. 2021 Aug 24;11:704919. doi: 10.3389/fcimb.2021.704919 (PMC8423026; doi:10.3389/fcimb.2021.704919)
Supplement: Supplementary file 8 [file Table_1.docx]

## Supplementary

Supplemental Figure 1


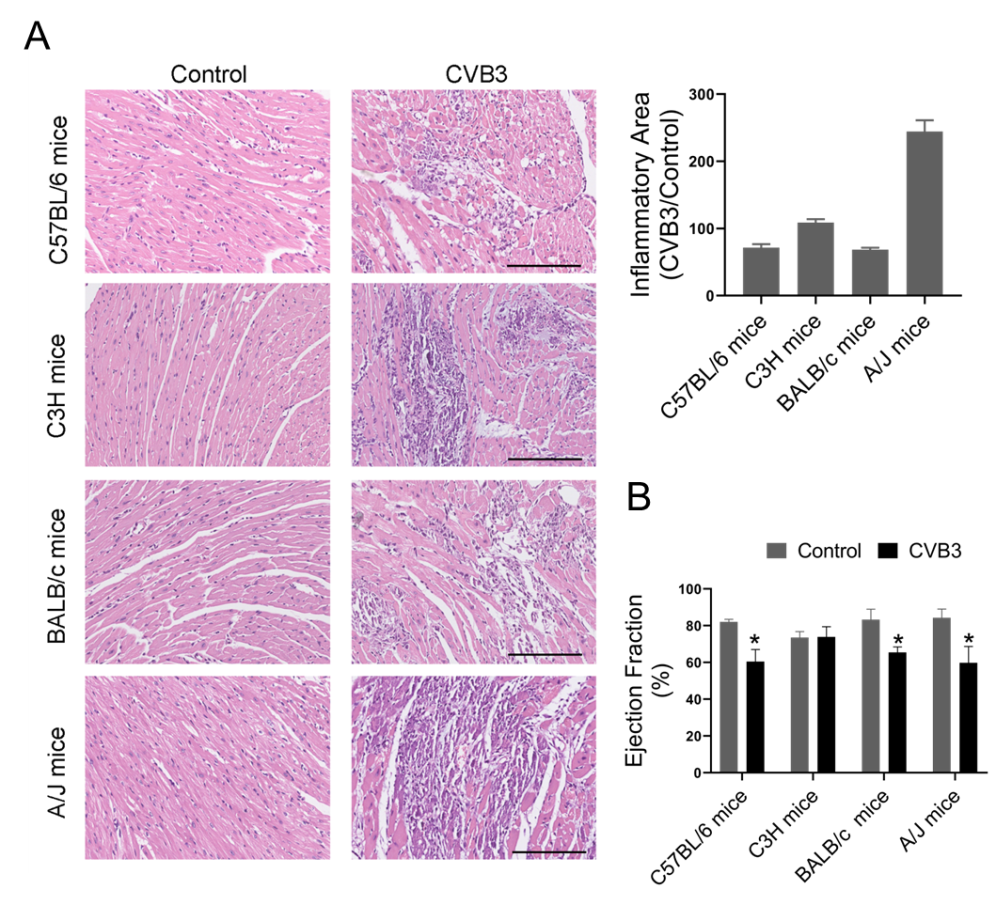


**Supplemental Figure 1. The validation of acute myocarditis mice models.** (A) The inflammatory infiltration in the heart tissues detected by H&E staining. (B) Cardiac function was analyzed by echocardiography; *P<0.05 VS. Control.

##
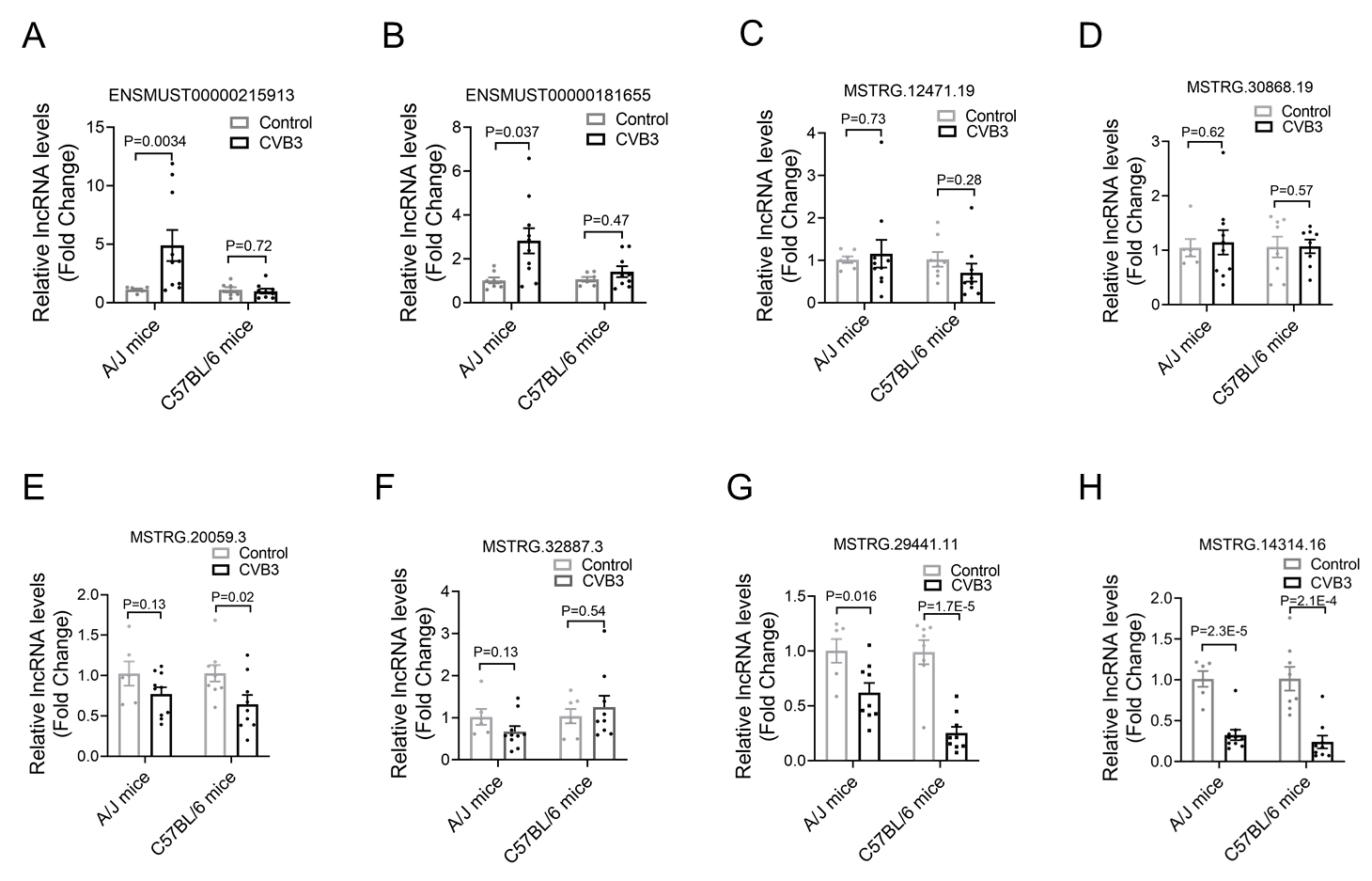
Supplemental Figure 2

**Supplemental Figure 2. The expression of lncRNAs in AM.** (A-H) The expression levels of lncRNAs in the heart tissues from CVB3-treated A/J mice and C57BL/6 mice were detected by qRT-PCR.

### Supplemental Figure 3

**
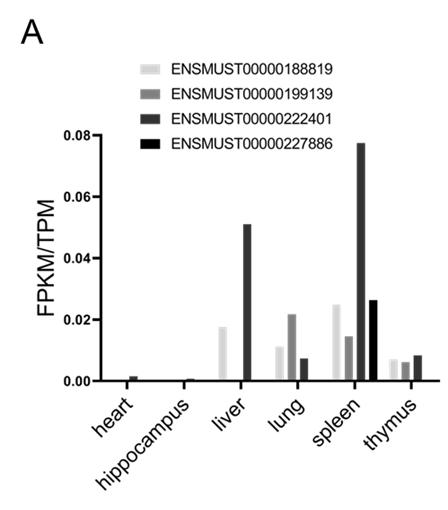
**

**Supplemental Figure 3. The expression of lncRNAs in mice organs.** (A) The expression levels of ENSMUST00000188819, ENSMUST00000199139, ENSMUST00000222401 and ENSMUST00000227886 in mice organs detected in NONCODE database.

| **Supplemental Table 1. Quantitative real-time PCR primers for lncRNA detection in mice.** | | |
| --- | --- | --- |
| **LncRNA IDs** | **Forward Primer (5’-3’)** | **Reverse Primer (5’-3’)** |
| ENSMUST00000199139 | GGCATTCTCGGAGGAAACCA | GACCAGAACAGCACGAGTCT |
| ENSMUST00000181655 | GCACACTGGAAACCAACGTC | CAGTACCTGGGTGGCTGAAG |
| ENSMUST00000188819 | CAGGAGTTCCTGGAGAGGGA | GTGCAGCCAGTTGGGAAAAG |
| ENSMUST00000227886 | CACCTGCTGCTGTAGTCCAA | GGAGATCCTGCAGCTTTGGT |
| ENSMUST00000215913 | CGGTCTACATACCCACTCGC | AGCCCTAGTTCAACCCAAGC |
| ENSMUST00000222401 | CCATGTGGCAGCCATAGTCT | ATCCTGAGGTGTGGGGTGTA |
| MSTRG.32887.3 | AAGCAGGAGTAGGTTGGGGA | AGAAGTGAGGCATGGAAGCA |
| MSTRG.20059.3 | ACCTTTCCGGAGGAGGCTTA | CCACAAAGGACGAGATCCCC |
| MSTRG.31357.2 | TCTGATGGGGGTCTCGTCTC | AGGCCACCGGACTCTTCTAA |
| MSTRG.30868.19 | CCCCCTGAGAATTTGGGGTC | GCTGACGGACTTTCCACAGA |
| MSTRG.31307.11 | TGGGAGATGAATGCCACTGC | CTTTCCAGCCTCCAAGGCA |
| MSTRG.12471.19 | CAACGGCACATGTGGTTTCC | GAAGCTGTGCAGTCCCTTCT |
| MSTRG.26098.49 | TCAGCGGGACCTAGGATTGA | GCAGGCTAGAATCTCGGACC |
| MSTRG.32881.28 | CCTCACCCACGAGTAACCAC | TGCCAAGTGTCCCTAGAGGT |
| MSTRG.29441.11 | TGATGGGATGCATTGGCCTT | TGGGGCTATGTCCCTCAGAA |
| MSTRG.32888.1 | ACAGGAAGGCCTAGGTCCAA | CAGACCCTTGGTGCTCTCAG |
| MSTRG.14314.16 | CTGTATGCAGTCTAGGGGCG | AGTCCCCTTTCTAGCCACCT |

### Supplemental Table 2. Fold changes of commonly dysregulated lncRNAs in AM.

| **LncRNA IDs** | **A/J mice** | **C57BL/6 mice** | **BALB/c mice** | **C3H mice** | **Cis-Targets Gene** | **Regulation** |
| --- | --- | --- | --- | --- | --- | --- |
| ENSMUST00000137154 | 6.50 | 7.39 | 6.19 | 4.41 | Cnp | up |
| ENSMUST00000188936 | 6.63 | 9.21 | 15.68 | 4.27 | Tenm3 | up |
| ENSMUST00000181085 | 7.54 | 20.92 | 4.67 | 13.32 | Exoc3l4 | up |
| ENSMUST00000132223 | 8.06 | 6.91 | 12.38 | 11.75 | Fbxo7 | up |
| ENSMUST00000219775 | 8.97 | 336.93 | 20.72 | Inf | Ifng | up |
| ENSMUST00000181742 | 9.87 | 13.82 | 11.10 | 4.92 | Zfp366 | up |
| ENSMUST00000181710 | 11.29 | 9.72 | 3.54 | 21.71 | Reep3 | up |
| ENSMUST00000138207 | 14.66 | 8.82 | Inf | Inf | Dnajc28 | up |
| ENSMUST00000207942 | 14.83 | 11.18 | 9.89 | 33.50 | Ccl7 | up |
| ENSMUST00000222401 | 15.87 | 17.28 | 13.51 | 163.76 | Dio3 | up |
| ENSMUST00000181655 | 16.70 | 14.31 | 4.92 | 76.24 | Lpp | up |
| ENSMUST00000227886 | 23.11 | 52.81 | 87.87 | 46.08 | Lrp12 | up |
| ENSMUST00000215913 | 30.12 | 40.06 | 25.13 | 81.79 | Gm49380 | up |
| ENSMUST00000188819 | 47.08 | 53.03 | 21.33 | 108.75 | Zbtb38 | up |
| ENSMUST00000199139 | 211.41 | Inf | 287.84 | 314.47 | Spp1 | up |
| ENSMUST00000189489 | 0.01 | 0.04 | 0.12 | 0.14 | H2bl1 | down |
| ENSMUST00000156387 | 0.07 | 0.08 | 0.13 | 0.20 | Plpp7 | down |
| ENSMUST00000236943 | 0.08 | 0.23 | 0.25 | 0.29 | Dusp1 | down |
| ENSMUST00000236043 | 0.09 | 0.02 | 0.18 | 0.06 | Nkx2-3 | down |
| ENSMUST00000196040 | 0.11 | 0.08 | 0.06 | 0.14 | Phgdh | down |
| ENSMUST00000181485 | 0.14 | 0.12 | 0.21 | 0.24 | Hes1 | down |
| ENSMUST00000143593 | 0.17 | 0.13 | 0.12 | 0.05 | Cobl | down |
| ENSMUST00000142815 | 0.17 | 0.21 | 0.22 | 0.24 | Gpbp1l1 | down |
| ENSMUST00000212695 | 0.18 | 0.20 | 0.26 | 0.11 | - | down |
| ENSMUST00000191207 | 0.21 | 0.08 | 0.04 | 0.07 | Gm28040 | down |
| ENSMUST00000192703 | -Inf | 0.00 | 0.12 | 0.11 | Morf4l1 | down |
| ENSMUST00000194907 | -Inf | 0.00 | 0.09 | 0.18 | Oprd1 | down |

| **Supplemental Table 3. Top 20 of specifically up- and down-regulated lncRNAs in FM mice.** | | | | |
| --- | --- | --- | --- | --- |
| **LncRNA IDs** | **Length (nt)** | **Cis-Targets Genes** | **Fold Change** | **Regulation** |
| ENSMUST00000209494 | 1252 | Spp1 | 211 | up |
| ENSMUST00000192087 | 1685 | Pfkfb3 | 304 | up |
| ENSMUST00000186326 | 959 | Lrrc46 | 314 | up |
| MSTRG.14905.35 | 11200 | Tcf4 | 376 | up |
| MSTRG.27034.4 | 1221 | Eid2b | 496 | up |
| MSTRG.7240.8 | 9535 | Lgmn | 577 | up |
| MSTRG.30868.33 | 9070 | Maml2 | 611 | up |
| MSTRG.21237.24 | 24603 | Pde4b | 702 | up |
| MSTRG.1193.1 | 6618 | Gpr39 | 808 | up |
| MSTRG.12863.1 | 1798 | Zfp52 | 1219 | up |
| MSTRG.32887.3 | 5259 | Fgf13 | 1314 | up |
| MSTRG.20059.3 | 8643 | Camk2d | 1631 | up |
| MSTRG.31357.2 | 1864 | Rbm7 | 1825 | up |
| MSTRG.30868.19 | 11668 | Maml2 | 2072 | up |
| MSTRG.31307.11 | 4574 | Mpzl2 | 2375 | up |
| MSTRG.12471.19 | 14549 | Cyyr1 | 2799 | up |
| MSTRG.30643.25 | 11368 | Cdh15 | 3410 | up |
| MSTRG.25917.47 | 7185 | Gm20696 | 5733 | up |
| MSTRG.14561.13 | 27882 | Fgf1 | 6983 | up |
| MSTRG.9506.19 | 6861 | Parg | 13398 | up |
| MSTRG.26098.49 | 35744 | Cacna1c | 3.2E-05 | down |
| MSTRG.32881.28 | 30232 | Fgf13 | 0.00011 | down |
| MSTRG.29441.11 | 9116 | Dlc1 | 0.00012 | down |
| MSTRG.32888.1 | 70288 | Fgf13 | 0.00015 | down |
| MSTRG.14314.16 | 9411 | Gm9955 | 0.00017 | down |
| MSTRG.2408.04 | - | Hivep2 | 0.00037 | down |
| MSTRG.18320.16 | 5322 | Gm11011 | 0.00038 | down |
| MSTRG.25361.3 | 5582 | Pde1c | 0.00042 | down |
| MSTRG.23146.49 | 33507 | Pcdh7 | 0.00044 | down |
| MSTRG.2409.2 | 9563 | Hivep2 | 0.00055 | down |
| MSTRG.13253.31 | 4464 | Ephx3 | 0.00068 | down |
| MSTRG.26383.5 | 4721 | Etv6 | 0.00084 | down |
| MSTRG.29854.3  MSTRG.9063.12 | 5643  6175 | Nr3c2  Depdc1b | 0.00095  0.0010 | down  down |
| MSTRG.14561.40 | 5008 | Fgf1 | 0.0012 | down |
| MSTRG.14481.83 | 12743 | Pcdhga10 | 0.0013 | down |
| MSTRG.3010.4 | 207 | - | 0.0016 | down |
| MSTRG.80.7 | 14405 | Prex2 | 0.0016 | down |
| MSTRG.20064.72 | 4892 | Zgrf1 | 0.0017 | down |
| MSTRG.31975.1 | 2707 | - | 0.0017 | down |
